# Epigenetic silencing of TIMP4 in heart failure

**DOI:** 10.1111/jcmm.12901

**Published:** 2016-07-11

**Authors:** Pankaj Chaturvedi, Suresh C. Tyagi

**Affiliations:** ^1^ Department of Physiology and BiophysicsSchool of MedicineUniversity of LouisvilleLouisvilleKYUSA

**Keywords:** heart failure, tissue inhibitor of matrix metalloprotease, epigenetic silencing

## Abstract

Tissue inhibitor of matrix metalloprotease 4 (TIMP4) is endogenously one of the key modulators of matrix metalloprotease 9 (MMP9) and we have reported earlier that cardiac specific TIMP4 instigates contractility and helps in differentiation of cardiac progenitor cells. Although studies show that the expression of TIMP4 goes down in heart failure but the mechanism is unknown. This study aims to determine the mechanism of silencing of TIMP4 in heart failure progression created by aorta‐vena cava (AV) fistula. We hypothesize that there is epigenetic silencing of TIMP4 in heart failure. To validate this hypothesis, we created heart failure model by creating AV fistula in C57BL/6 mice and looked into the promoter methylation (methylation specific PCR, high resolution melting, methylation sensitive restriction enzyme and Na bisulphite treatment followed by sequencing), histone modification (ChIP assay) and microRNAs that regulate TIMP4 (mir122a) and MMP9 (mir29b and mir455‐5p). The physiological parameters in terms of cardiac function after AV fistula were assessed by echocardiography. We observed that there are 7 CpG islands in the TIMP4 promoter which get methylated during the progression of heart failure which leads to its epigenetic silencing. In addition, the up‐regulated levels of mir122a in part, contribute to regulation of TIMP4. Consequently, MMP9 gets up‐regulated and leads to cardiac remodeling. This is a novel report to explain the epigenetic silencing of TIMP4 in heart failure.

## Introduction

What makes tissue inhibitor of matrix metalloprotease 4 (TIMP4) interesting is that TIMP4 can inhibit matrix metalloprotease 9 (MMP9) without activation and within the heart it is the endogenous inhibitor of MMP9. Overexpression of TIMP4 in the myocardial infarction (MI) hearts reduces the levels of MMP9 1. We discovered earlier that intraperitoneal administration of TIMP4 (cardiac inhibitor of metalloproteinase) conferred cardioprotection and mitigated oxidative stress 2. The activation of MMP9 beyond a certain limit is deleterious to the heart and our laboratory has adequately shown that MMP9 activation worsens cardiac remodeling 3, 4, 5, 6, 7. The hyper activation of MMP9 represents the failure of the endogenous TIMP4 to inhibit MMP9 within time and studies have shown that there is down‐regulation of TIMP4 in heart failure 1, 2. Mice which are deficient in TIMP4 have poor regeneration capacity and fail to recover fully from MI 8. Previous study from our laboratory has demonstrated that TIMP4 plays an important role in myocyte contractility and differentiation of cardiac stem cells to cardiomyocytes 9. Exogenous supply of TIMP4 or its overexpression in heart is cardioprotective 1, 2. In this study, we speculate that there is epigenetic silencing of TIMP4 in heart failure which allows the MMP9 to increase. This study is based on our previous finding that the mRNA levels of TIMP4 are down‐regulated in AV fistula hearts 10 with activation of MMP9. Elevated activation of MMP9 has been reported by our laboratory to cause cardiac fibrosis, altered collagen/elastin ratio, myocyte‐myocyte uncoupling by degrading connexins and oxidative stress 3, 11, 12, 13, 14.

The evaluation of the TIMP4 promoter sequence (GeneBank: AY072631.1) using the methprimer site (http://www.urogene.org/cgi-bin/methprimer/methprimer.cgi) suggested that there are seven CpG islands in the TIMP4 promoter sequence. We hypothesize that in heart failure these CpG islands get methylated which leads to the silencing of the TIMP4 gene. In our previous study 9, we discovered that when TIMP4 is expressed in myocytes there is decrease in the expression of microRNA122a along with increased levels of serca2a. In this study, we evaluated whether diminished expression of TIMP4 is affected by mir122a. We also evaluated the expression of mir29b, and mir455‐5p which we have reported to regulate the expression of MMP9 6. Although, there are no studies in heart that explain epigenetic silencing of TIMP4 due to methylation, but there is a report in lung cancer which shows that CpG islands in the TIMP4 promoter region get methylated 15. In most of the lung cancer cell lines there was frequent methylation of the CpG islands for TIMP4 (64%). Similarly, CpG methylation has also been observed in TIMP2 and TIMP3 though not in heart 16, 17, 18, 19. These studies do suggest that in diseased condition there is imbalance of MMP: TIMP axis, particularly the MMP up‐regulation that leads to extracellular matrix remodeling which is accompanied by epigenetic silencing of TIMPs. Apart from methylation of these genes, there are other mechanisms that underline the silencing of these genes. Qin and Han 20 have reported that histone deacetylase 4 (HDAC4) mediates repression of matrix metalloproteases in tissue fibrosis. In this study, we evaluated HDAC1 as well as histone acetylation at lysine 9. We also evaluated the expression of DNA methyltransferase 1 (DNMT1) to check if hypermethylation of TIMP4 is associated with higher expression of DNMT1. This study clearly demonstrates that the down‐regulation of TIMP4 is due to methylation of the CpG islands in the promoter region of TIMP4. In addition, mir122a is also up‐regulated in volume overload heart failure which contributes to its epigenetic silencing.

## Material and methods

### Animals and experimental design

For animal care and health, standard procedures as per guidelines of the National Institute of Health were followed and approved by the Institutional Animal Care and Use Committee, University of Louisville. Volume overload was created in the wild‐type C57BL/6J mice (~80 days old male) by aorta‐vena cava fistula (AVF), as described by our earlier study 10. Briefly, the mice were anaesthetized using 5% isoflurane in 100% oxygen (flow rate 1 l/min.) and maintained anaesthesia using 1–3% isoflurane. The caudal vena cava ~1 cm below the renal arteries was identified. The overlying adventitia was removed by blunt dissection to expose the vessels, taking care not to disrupt the tissue connecting the vessels. Both vessels were occluded proximal and distal to the desired puncture site. An 18 guaze needle was inserted into the exposed abdominal aorta and advanced through the medial wall into the vena cava to create the AV fistula. The needle was withdrawn and the ventral aortic puncture was sealed with tissue glue (3M vetbond). The pulsatile flow of oxygenated blood from aorta to the vena‐cava showed successful AV fistula. The skin incision and abdominal musculature was closed with standard absorbable sutures. The mouse was kept warm and monitored for 2 hrs and then transferred to the animal care facility. Sham surgery was performed in the same manner except no puncture was made and referred to as WT. The cardiac function was evaluated by echocardiography and tissues were collected after 6 weeks of AVF and evaluated for the expression of genes. Before collecting the heart tissues, all the blood was removed from the heart with the help of a syringe and the heart was perfused free of blood with PBS. For immunohistostaining, the heart was immobilized in freezing media and kept at −20 for cryosectioning. For RNA and protein extraction, the tissue was frozen in liquid nitrogen and kept at −80 till further use.

### Echocardiography

Echocardiography was done using the VIVO 2100 system (Visual Sonics, Toronto, ON, Canada) for evaluating the cardiac function. The mouse was positioned supine on a heating table (37°C) under isoflurane inhalation after anaesthetizing with isoflurane. Hair from the chest area was removed by hair removal cream (Nair) and acoustic gel (Other‐Sonic, Pharmaceutical Innovations, Newark, NJ, USA) was applied to perform imaging using the transducer (13–24 MHz) in both long and short axis. The measurements were performed in B‐mode and M‐mode with the transducer which was held immobile by integrated rail system. The cine loops were analysed for left ventricular posterior wall, diastole (LVPWd), left ventricular internal diameter, diastole (LVIDd), ejection fraction and fractional shortening.

### Haematoxylin and eosin staining

Haematoxylin and eosin staining was performed by Rapid Chrome haematoxylin and eosin staining kit (Thermo Scientific, Waltham, MA, USA) as per manufacturer's instructions. Briefly, the paraffin sections were hydrated and placed in Rapid Fix for 5–7 sec. The slides were washed with distilled water and placed in haematoxylin for 1 min. The slides were placed in Bluing Reagent (three dips) after washing with distilled water. The slides were washed in 95% alcohol (5–7 dips) and placed in Eosin‐Y for 15 sec. The slides were again washed with 95%, 100%, alcohol (5–7 dips) and placed in xylene (5–7 dips). The slides were mounted using the Shandon mounting medium and observed under visible microscope (×20 objectives; QCapture Pro, Surrey, BC, Canada).

### Masson's trichrome staining

Masson's trichrome staining was performed with Chromaview Masson's trichrome staining kit (Thermo Scientific) as per manufacturer's instructions. Briefly, the paraffin sections were hydrated and placed in Bouin's Fluid at 56°C for 1 hr. The sections were rinsed in tap water for 5 min. and placed in Working Weigert's Iron Haematoxylin Stain (Weigert's Iron Haematoxylin A+ Weigert's Iron Haematoxylin B) for 10 min. After washing in tap water for 10 min., the sections were placed in Biebrich Scarlet Acid Fuchsin solution for 7 min. The slides were again washed in tap water and placed in Phosphotungstic Phosphomolybdic Acid solution for 5 min. The slides were stained in Aniline Blue Stain solution for 7 min. and placed in 1% Acetic Acid solution for 1 min. The slides were rinsed in distilled water and dehydrated in anhydrous alcohol for 1 min. each (70%, 90% and 100%). The slides were placed in xylene for three times, 1 min. each and mounted. The slides were observed using bright field microscopy (×20 objectives; QCapture Pro).

### Promoter methylation analysis

The sequence of the promoter region was taken from NCBI data base: *Mus musculus* TIMP4 gene; promoter region, exon 1 and partial cds GenBank: AY072631.1. The methylated and unmethylated primers were designed using the Methprimer website and primers with at least 4 CpG's in the product were selected for PCR amplification of the sodium bisulphite treated DNA. The genomic DNA was isolated from the AVF and WT mice hearts using the DNA isolation kit (27220 Turnberry Lane Suite 200; Qiagen, Valencia, CA, USA) and subjected to sodium bisulphite treatment using the EZ‐DNA methylation kit (Zymo Research Corporation, Irvine, CA, USA). The treated DNA was PCR amplified using the methylated/unmethylated primers and subjected to Sanger DNA sequencing.

### Methylation sensitive restriction enzyme analysis

For methylation sensitive restriction enzyme analysis (MSRE), the genomic DNA (1 μg) was digested with *HaeII* and PCR amplified with MSRE primers designed from the promoter region of the TIMP4 gene. If methylation is present, the DNA is not digested and we get the PCR band that corresponds to 1.267 kb on the other hand in the absence of methylation, DNA is digested and no amplification is observed.

### MS PCR and high resolution melting analysis

We performed methylation specific PCR from the sodium bisulphite treated DNA of both WT and AVF mice. We used methylated and unmethylated primers designed from the methprimer website. We performed high resolution melting analysis using the methylated and unmethylated primers and the sodium bisulphite treated genomic DNA. We used LightCycler^®^ 480 High Resolution Melting Dye in the Light cycler 480 system (Roche Diagnostics Corporation, Indianapolis, IN, USA) as per manufacturer's instructions. We followed the protocol as described by Krypuy *et al*. 21. The melting curves showed a shift with the methylated primer, which represents higher methylation of the promoter region of the TIMP4 gene.

### Immunohistochemistry

The immobilized tissues in the freezing media were cryosectioned using Cryostat (Leica CM1850; Leica, Buffalo Grove, IL, USA) and fixed using paraformaldehyde. The tissues were permeabilized using Triton X 100 (0.3%) and Primary antibodies were added at 1:250 dilution. The tissues were incubated in the primary antibody overnight at 4°C and next day washed with Tris buffered saline (TBS) and Tris buffered saline with Tween 20 (TBST). The slides were tagged with appropriate fluorescently tagged secondary antibodies, and visualized with laser scanning confocal microscope (Olympus FluoView1000, Olympus America Inc., Melville, NY, USA).

### Western blotting

The protein was extracted from the heart tissues using Radio Immuno Precipitation Assay Buffer (RIPA) buffer containing Phenylmethylsulfonyl fluoride (PMSF) and protease inhibitor and quantitated by Bradford method using spectramax M2 (Molecular Devices, Sunnyvale, CA, USA). The protein samples were prepared in SDS sample buffer (2% SDS, 10 mM dithiothreitol, 60 mM Tris‐HCl pH 6.8, bromophenol blue 0.1%) and loaded on 10% PAGE. The samples were electro‐transferred to PVDF membrane and probed with appropriate primary and secondary Horse Radish Peroxidase (HRP) conjugated antibodies. The membrane was developed with the Western blotting detection system ECL (GE Healthcare, Piscataway, NJ, USA) and imaging was done using the gel documentation system ChemiDoc XRS system (Bio‐Rad, Richmond, CA, USA). After stripping, the membranes were reprobed with anti‐GAPDH antibody (Millipore, Billerica, MA, USA) as control. Image Lab densitometry software (Bio‐Rad) was used to analyze the data and normalizing the GAPDH. The description of antibodies is: (*i*) TIMP4‐ ~26 kD (Abcam, Cambridge, MA, USA) (ab58425), (*ii*) MMP9 ~92 kD Abcam (ab38898), (*iii*) DNMT1 ~100 kD Abcam (ab13537), (*iv*) histone 3 acetylation at lysine 9 (H_3_K9Ac) Abcam (ab10812) molecular wt ~18 kD.

### RT‐PCR and real time PCR

Total RNA was extracted using the Trizol reagent (Life Technologies, Carlsbad, CA, USA) and microRNA was extracted using the mirVANA RNA isolation kit (Life Technologies) according to the manufacturer's instructions. For evaluating the mRNA expression of TIMP4, cDNA was synthesized using the High Capacity cDNA Reverse Transcription kit (Applied Biosystems, Foster City, CA, USA). Real time PCR was performed with the primers Forward 5′‐TCTGAACTGTGGCTGCCAAAT and Reverse 5′‐AGCTTTCGTTCCAACAGCCAG‐3′ using the Sybr green kit (Quanta, Gaithersburg, MD, USA) using manufacturer's instructions. For microRNAs, the cDNA was synthesized using the miscript II RT kit (Qiagen) as per manufacturer's instructions. Real time PCR was performed with the Rnu6, mir122a, mir29b and mir455‐5p primer assays (Qiagen) in the Stratagene Mx3000P real time PCR machine. For calculating the fold expression, delta C_t_ method was used after normalizing the genes with 18s rRNA or Rnu6.

### Gelatin zymography

Gelatin zymography was performed as stated earlier 22. Briefly, 30 μg of the heart extract was incubated with SDS sample buffer (without reducing agents) at 37°C for 30 min. and loaded on 10% SDS‐PAGE gel prepared with 2% gelatin. After the proteins got separated, the gel was washed three times with 2.5% Triton X‐100 for 20 min. each to remove SDS. The gel was then incubated for 24 hrs at 37°C in activation buffer (5 mmol/l Tris HCl‐pH 7.4, 0.005% v/v Brij‐35, and 1 mmol/l CaCl_2_). The gel was stained in comassie to observe clear bands that appear as a result of proteolytic acitivity of MMPs for gelatin. The gel was documented on gel documentation system (Bio‐Rad, Hercules, CA, USA) and analyzed with image lab software (Bio‐Rad, Hercules, CA).

### *In situ* zymography

*In situ* zymography was performed for heart tissue sections using DQ gelatin (Molecular Probes, Grand Island, NY, USA) as per manufacturer's instructions. Briefly, the cryosectioned heart tissue was incubated at RT and all the media was removed. The sections were washed with PBS for 5 min., air‐dried and overlayed with DQ gelatin (Molecular Probes). The slides were incubated for 2 hrs and then washed in PBS for 5 min. The slides were dried and added mounting media then covered with cover slip. The slides were observed in confocal microscope (Olympus FluoView1000) at 488 nm.

### Chromatin immunoprecipitation

For chromatin immunoprecipitation (ChIP) assay we followed the protocol as described earlier 22. Briefly, we used the Abcam kit as per manufacturer's instructions. The tissue was first fixed with paraformaldehyde and lysed with buffers A and B. The lysate was centrifuged to remove the supernatant and resuspended in buffer C. The resulting DNA was sonicated (sonic dismembrator model 100; Fisher Scientific, Waltham, MA, USA) to produce DNA fragments of size 200–1000 bp. The sheared DNA was incubated with H_3_K9Ac antibody provided with the kit overnight and mixed with beads for immunoprecipitation. The DNA was then purified and checked with PCR using the forward primer GCAATGATGTGCAGTAGGCG and reverse primer GCAACAGCAAACAGTCAGGG.

### Statistical analysis

All the data analysis was performed with SPSS 16.0 (SPSS Inc., Chicago, IL, USA) and presented as mean ± S.E.M. unless stated otherwise. We compared two groups by using by Student's independent *t*‐test and *P* < 0.05 was considered as significant for *n* = 6.

## Results

### There is decreased cardiac function in AVF mice

**Figure 1:**
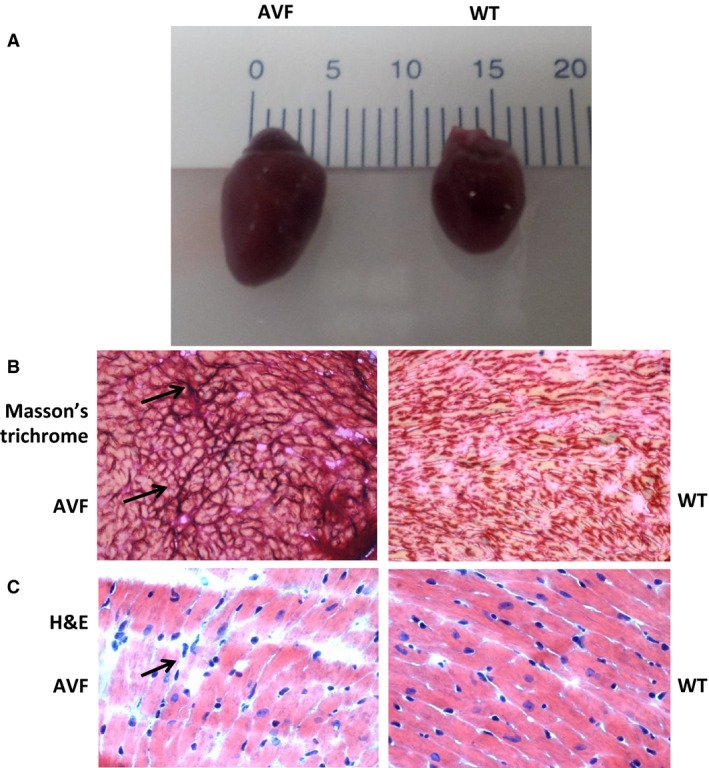
Dysregulation of cardiac structure in AVF mice. (**A**) The hearts of Aorta‐vena cava fistula (AVF) mice are enlarged as compared to the wild‐type (WT) mice due to volume overload in the right ventricle. (**B**) Masson's trichrome staining shows that there is more collagen deposition in AVF mice as compared to WT (arrows). (**C**) Haematoxylin and eosin staining shows that the structure of myocytes is dysregulated in AVF mice (arrows), which suggests that there is myocyte uncoupling that eventually leads to heart failure.

**Figure 2:**
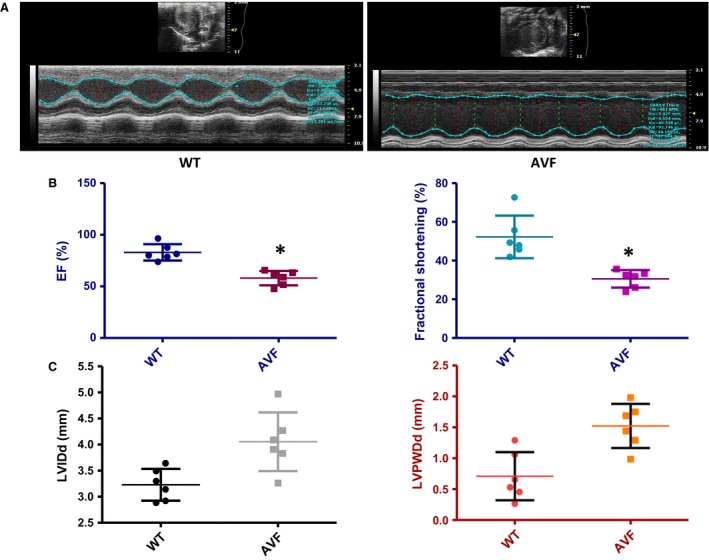
Decreased cardiac function in AVF mice. There is decreased cardiac function in AVF mice as compared to WT mice. (**A**) Echocardiograph of AVF and WT mice hearts. (**B**) Graphs representing ejection fraction and fractional shortening. Decrease in ejection fraction is a hallmark of volume overload model which is accompanied by fractional shortening. There is a significant decrease in the ejection fraction and fractional shortening in AVF mice as compared to WT mice (*P < 0.05, ±S.D. for AVF *versus*, WT Student's *t*‐test, *n* = 6). (**C**) Graphs representing left ventricular internal diameter, diastole (LVIDd) and left ventricular posterior wall, diastole (LVPWd). There is increase in the LVIDd and LVPWd in AVF mice as compared to WT mice. The increase in internal diameter of ventricle is a hallmark of hypertrophy.

We observed cardiac hypertrophy (Fig. 1A) in AVF mice with increased collagen deposition as evident from Masson's trichrome staining (Fig. 1B). The haematoxylin and eosin staining (Fig. 1C) showed that in AVF mice the myocyte structure is dysregulated which suggests uncoupling of the myocytes from the matrix and myocyte‐myocyte uncoupling. We evaluated cardiac function in the AVF and WT mice using the Vevo 2100 (Visual Sonics) system. We observed a significant decrease in the ejection fraction (Fig. 2A and B) and fractional shortening (Fig. 2B) in AVF mice as compared to the WT mice, which is a hallmark of volume overload. In addition, there was increase in the internal diameter of the ventricle as evident from the LVIDd and LVPWDd (Fig. 2C), which suggests cardiac hypertrophy. The results suggested decrease in the cardiac function in AVF mice.

### TIMP4 promoter is methylated at the CpG sites

**Figure 3:**
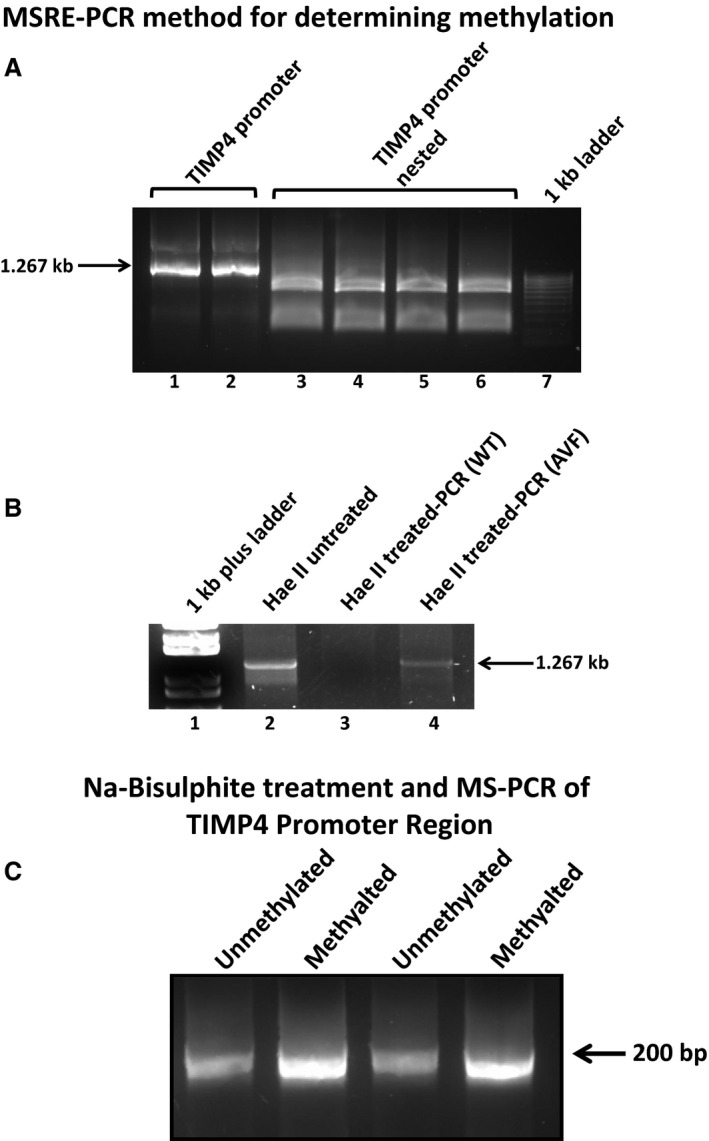
Methylation specific restriction enzyme analysis. (**A**) The amplification of the TIMP4 promoter showed 1.26Kb band (first two lanes) which was confirmed by nested primers (lanes 3–6). (**B**) Methylation specific restriction enzyme analysis. The genomic DNA from WT and AVF mice were digested with *HaeII* and amplified using TIMP4 promoter primers. Lane 1 shows marker, Lane 2 shows untreated DNA amplified with TIMP4 promoter primers. Lanes 3 and 4 show WT and AVF DNA respectively, treated with *HaeII* and amplified with TIPM4 promoter primers. (**C**) Na bisulphite treatment and MS‐PCR of TIMP4 Promoter Region was done with methylated and unmethylated primers designed from the CpG islands of the promoter region.

**Figure 4:**
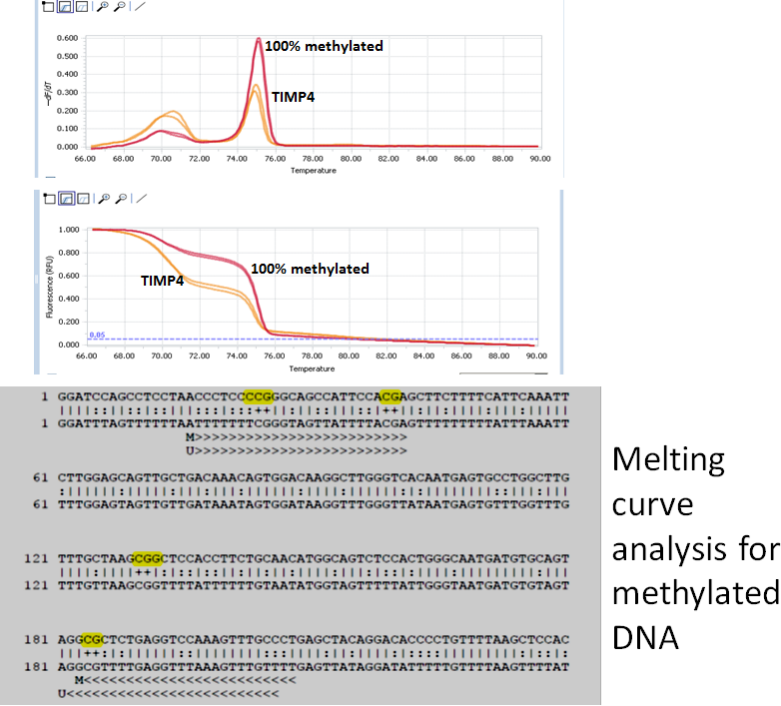
High resolution melting for methylation analysis. Na bisulphite treated DNA from the heart tissues was used for MS‐HRM analysis using the primers designed from the methprimer site along with appropriate controls as per manufacturer's instructions (Resolite, Light cycler 480 system Roche, 21). Cpgenome Universal Methylated DNA (Chemicon, Milipore) was used as 100% methylated control DNA. The reaction was set up with the Na bisulphite treated DNA, Control DNA, primers and the resolute mixture provided with the kit. The HRM program was used in the Light cycler 480 system for the amplification. After amplification, the data were analysed using the inbuilt software. The peak in red shows the positive control while yellow peak shows the TIMP4 promoter. The yellow peak shows ~50% methylation at the TIMP4 promoter site to encompass the CpG islands.

**Figure 5:**
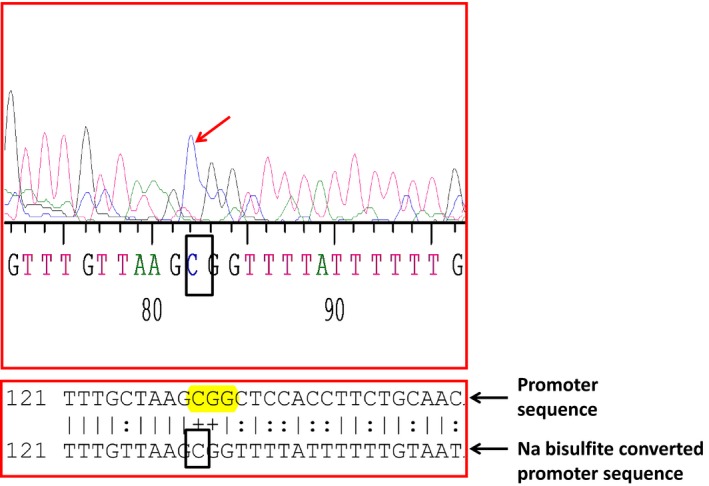
Confirmation of the methylation at CpG island by sequencing: The Na bisulphite treated DNA was PCR amplified and sequenced to confirm the methylation at the CpG islands. The arrow shows the peak for C which is methylated and not converted to T after Na bisulphite treatment.

There are seven CpG islands in the promoter region (Fig. S2) as indicated by the methprimer website. We found three sites for *HaeII* restriction enzyme for methylation specific restriction digestion (Fig. S3). The primers for MSRE analysis showed a PCR product of 1.26 kb which was confirmed with nested primers (Fig. 3A). The genomic DNA from WT and AVF mice was digested with *HaeII* and PCR amplified using the TIMP4 promoter primers. In the untreated control DNA, we observed a PCR product of 1.26 kb while in the WT DNA, we did not observe any amplification which suggests that WT DNA is not methylated and digested by the HaeII enzyme. We got amplification with AVF DNA, which suggests methylation in the TIMP4 promoter region (Fig. 3B). The MS‐PCR of the sodium bisulphite treated DNA using methylated and unmethylated primers showed higher amplification with methylated primers as compared to unmethylated in the AVF mice (Fig. 3C). The high resolution melting (HRM) analysis using the Roche 480II cycler showed a shift in Tm with the methylated primer as compared to the unmethylated primer (Fig. 4). After treatment of the genomic DNA with sodium bisulphite, the PCR products were sequenced and we confirmed that the CpG islands were methylated in the promoter region (Fig. 5). All these results suggest that there is increased methylation in the promoter region of the TIMP4 gene.

### Down‐regulation of TIMP4 in the AVF mice

**Figure 6:**
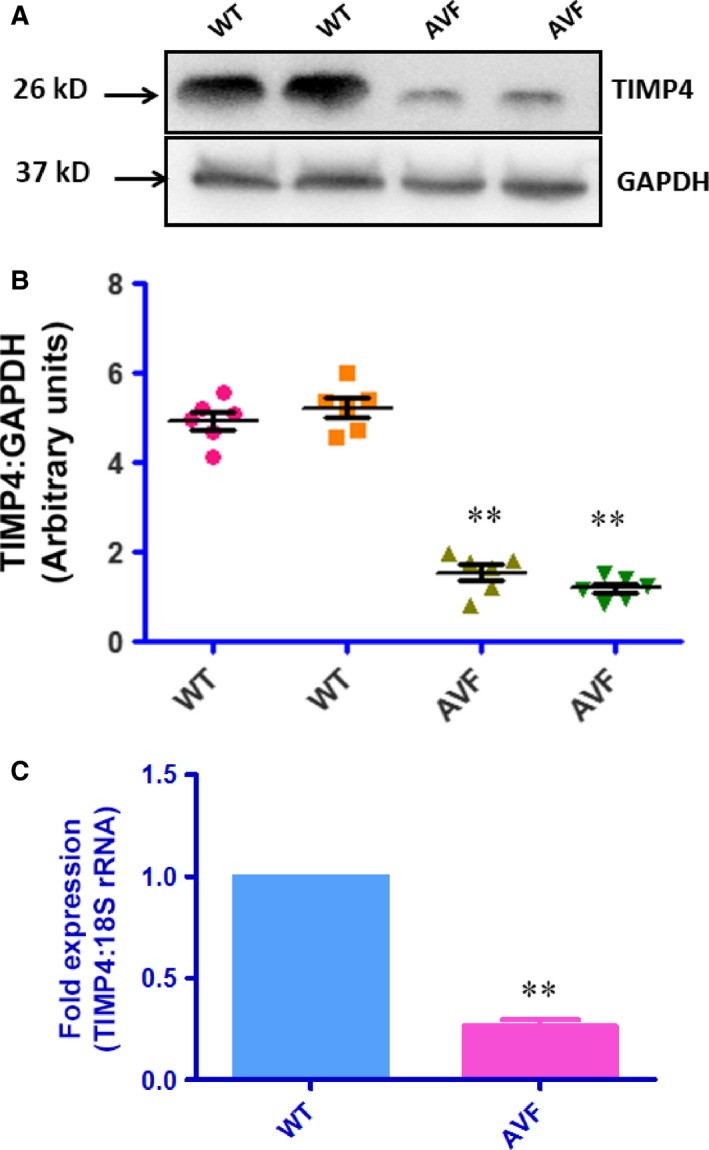
(**A**) TIMP4 goes down in heart failure. Western blot of heart tissues from WT and AVF mice show that TIMP4 goes down in AVF mice (**P < 0.01, ±S.E.M., AVF *versus* WT, Student's *t*‐test, *n* = 6). (**B**) Real time PCR for the TIMP4 mRNA levels. We observed Five fold decrease in the mRNA levels in AVF mice as compared to WT mice (**P < 0.01, ±S.E.M., AVF *versus* WT).

**Figure 7:**
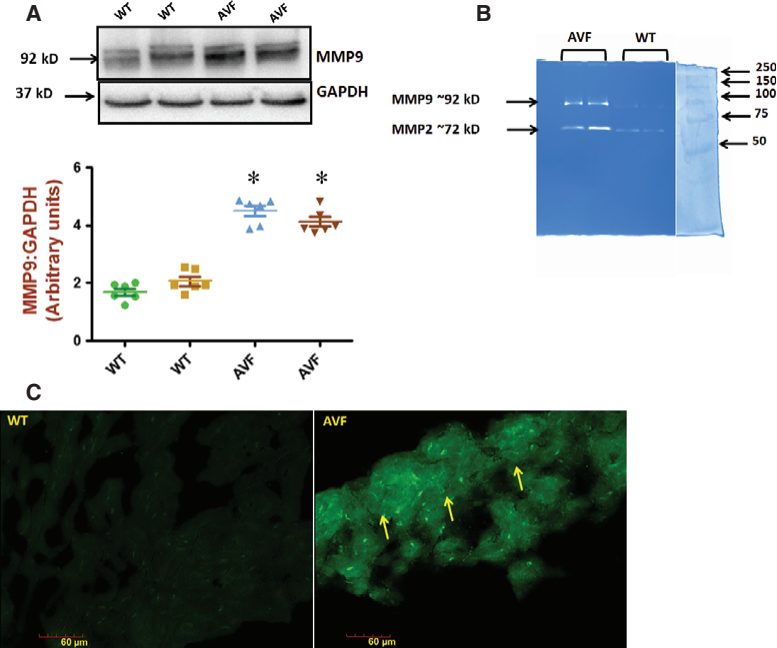
MMP9 is up‐regulated in heart failure. (**A**) MMP9 is up‐regulated in heart failure as shown by Western blot. (**B**) MMP zymography (*P < 0.05, ±S.E.M., AVF *versus* WT, Student's *t*‐test, *n* = 6). The zymography gel shows two bands of MMP9 (~92 kD) and MMP2 (~72 kD). The extreme right lane shows the marker (Precision Plus Protein Standards, Bio‐Rad) and it was loaded in the same gel as the samples and photographed before staining. (**C**) *In situ* zymography. *In situ* zymography was performed with DQ gelatin (Molecular Probes) as per manufacturer's instructions. The DQ gelatin was overlayed on the heart sections and incubated for 2 hrs to get the total MMP activity in the form of green fluorescence at 488 nm. The right panel shows the AVF heart with MMP activity while the WT heart in the left panel does not show any MMP activity.

**Figure 8:**
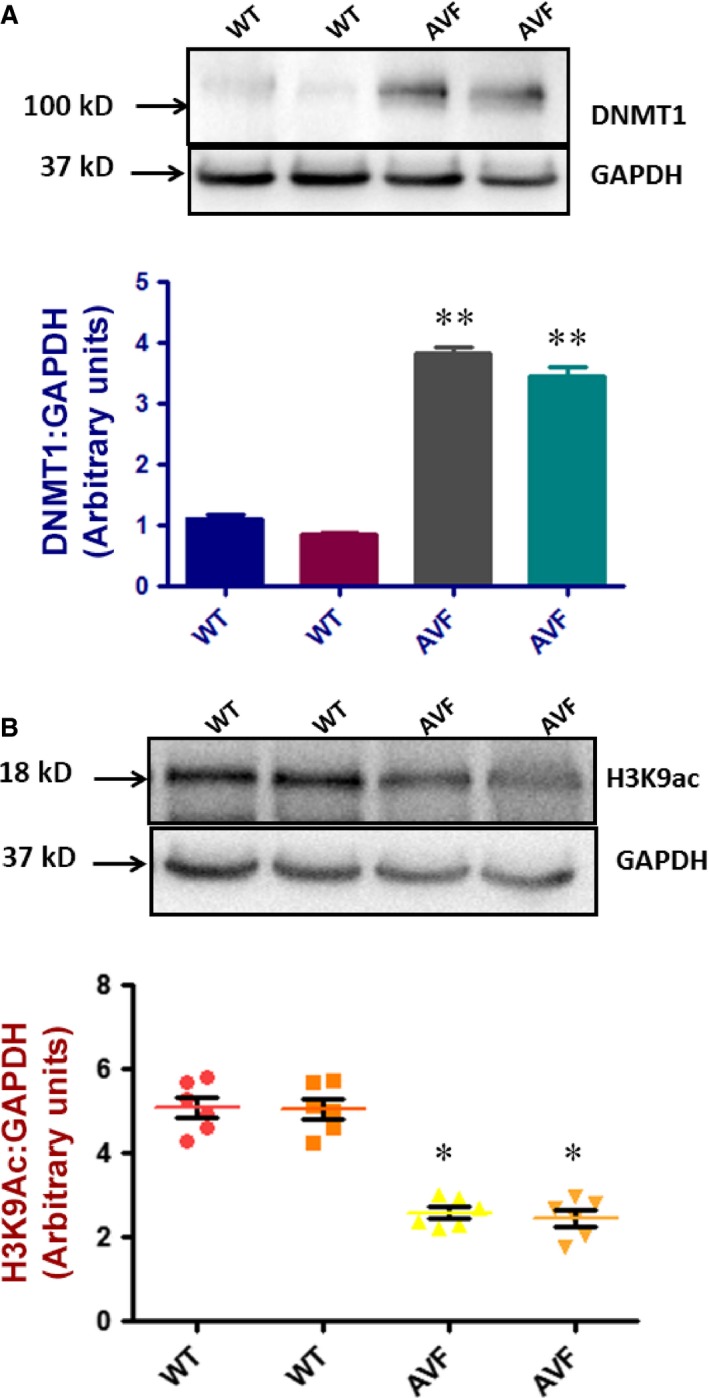
DNMT1 is elevated in heart failure. (**A**) Western blot show that DNMT1 is up‐regulated in heart failure (**P < 0.01, ±S.E.M., AVF *versus* WT, Student's *t*‐test, *n* = 6). (**B**). Decrease in acetylation. There is a decrease in acetylation in heart failure of histone 3 at lysine 9 position as represented by Western blot (*P < 0.05, ±S.E.M., AVF *versus* WT, Student's *t*‐test, *n* = 6).

**Figure 9:**
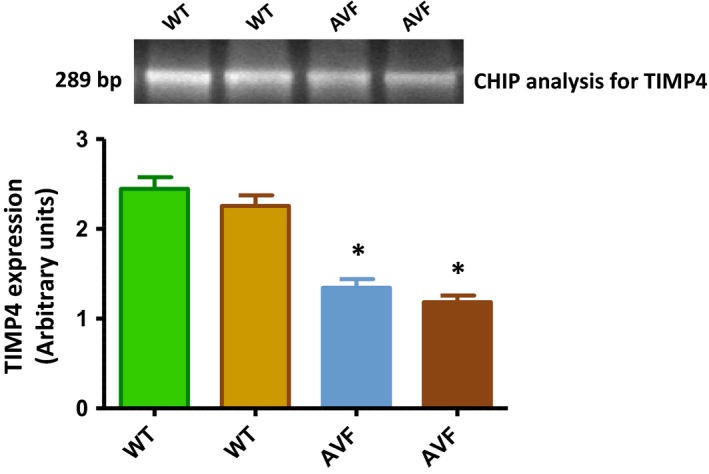
Chromatin immunoprecipitaiton assay. The genomic DNA from WT and AVF mice was sheared and immunoprecipitated using the antibody provided in the kit (abcam CHIP). The precipitate was then PCR amplified with TIMP4 promoter primers to check the histone acetylation in the promoter region. The results suggest a decrease in the histone acetylation (*P < 0.05, ±S.E.M., AVF *versus* WT, Student's *t*‐test, *n* = 6).

We evaluated the expression of TIMP4 in both WT and AVF mice with Western blots and real time PCR and found down‐regulated expression in the AVF mice as compared to the WT mice (Fig. 6A and B). Correspondingly, the expression of MMP9 was up‐regulated as confirmed by Western blots (Fig. 7A). The activity of MMP9 and MMP2 was confirmed with gelatin zymography which was found to be up‐regulated (Fig. 7B). The activity of total MMPs was also checked by *in situ* gelatin zymography which was found to be up‐regulated (Fig. 7C). To check whether down‐regulated levels of TIMP4 is due to involvement of DNMT 1, which is a DNA methyl transferase, we evaluated DNMT1 levels with Western blots. We found up‐regulated levels of DNMT1 (Fig. 8A) in the AVF heart tissues with Western blots. We observed down‐regulated levels of histone3 acetylation at lysine9 which suggests that there is decrease in the acetylation of histone at lysine 3 (Fig. 9B). All these results indicate that there is epigenetic silencing of TIMP4 and since native TIMP4 is an active inhibitor of MMP9, there was augmented level of MMP9.

### TIMP4 promoter is more deacetylated in AV mice

Chromatin immunoprecipitation for the histone acetylation at the TIMP4 promoter region showed down‐regulated levels of TIMP4 in the precipitated DNA from AVF mice as compared to WT (Fig. 9). This suggests that methylation is not the only factor that is responsible for down‐regulation of TIMP4. The down‐regulated histone acetylation also contributes to this effect.

### TIMP4, DNMT1 and HDAC‐1 have altered protein levels

**Figure 10:**
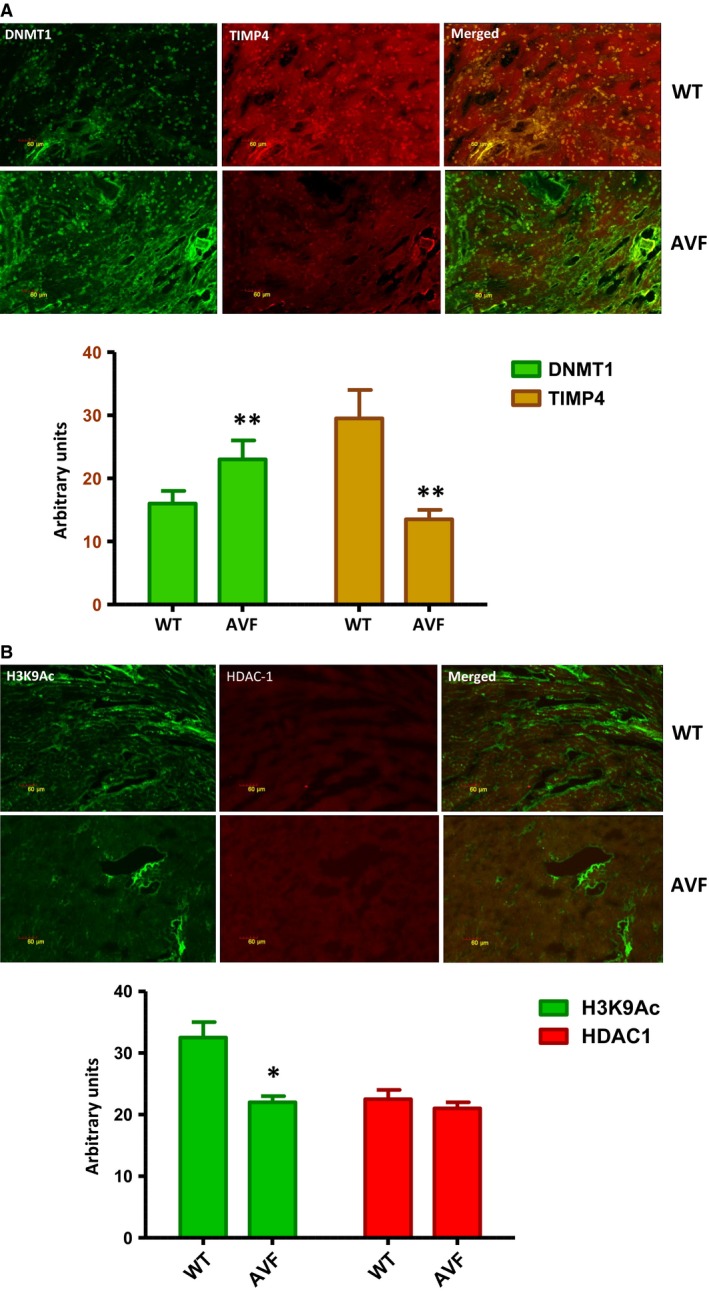
Immunohistostaining. (**A**) Immunohistostaining showed that there is down‐regulation of TIMP4 in the heart tissues of AVF mice as compared to WT mice (**P < 0.01, ±S.E.M., AVF *versus* WT, Student's *t*‐test, *n* = 6). (**B**) There was down‐regulation of H3K9Ac in the AVF mice as compared to WT although we observed no change in the HDAC‐1 (*P < 0.05, ±S.E.M., AVF *versus* WT, Student's *t*‐test, *n* = 6).

Immunohistochemistry of the heart tissue from WT and AVF mice showed down‐regulation of TIMP4 in the heart of AVF mice (Fig. 10A). There was up‐regulation of DNMT1 which was in accordance with the Western blot results (Fig. 10A). Although there was down‐regulation of H_3_K9Ac (Fig. 10B), the HDAC1 levels did not change in the AVF mice showing that the histone deacetylation is not affected in the AVF mice (Fig. 10B).

### Real time expression of mir122a, mir29b and 455‐5p

**Figure 11:**
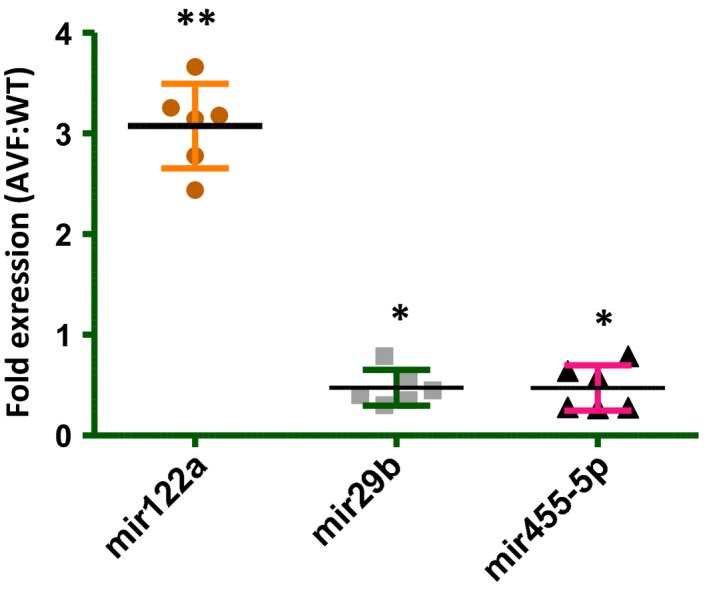
MicroRNA expression. We checked the expression of microRNAs 122a which we reported earlier to be involved in TIMP4 regulation and mir29b and mir455‐5p, which were reported to be involved in MMP9 regulation. We found up‐regulated expression of mir122a (**P < 0.01, ±S.E.M., AVF *versus* WT, Student's *t*‐test, *n* = 6) and down‐regulated expression of mir29b and mir455‐5p in AVF mice as compared to WT (*P < 0.05, ±S.E.M., AVF *versus* WT, Student's *t*‐test, *n* = 6).

**Figure 12:**
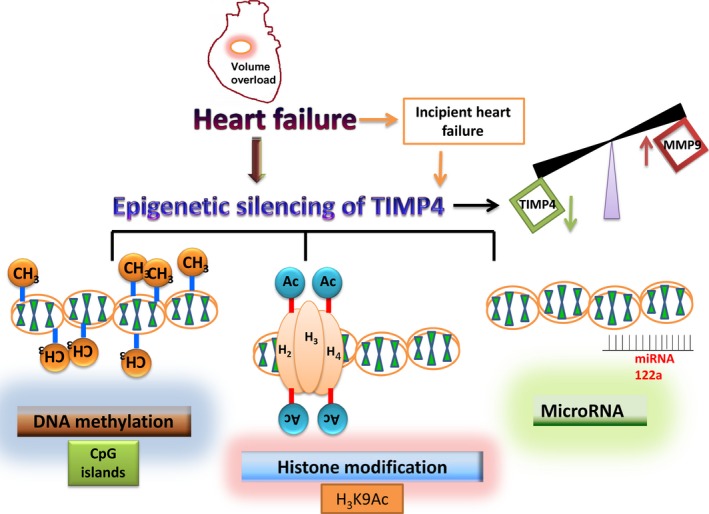
Overall schematic of epigenetic silencing of TIMP4 in heart failure.

We performed real time expression of three microRNAs‐122a, 29b and 455‐5p since we found in our previous reports that mir29b and 455‐5p regulate the levels of MMP9 6 and mir122a is associated with TIMP4 expression 9. We observed up‐regulated expression of mir122a and down‐regulated expression of mir29b and 455‐5p (Fig. 11). We have represented the overall picture of epigenetic silencing of TIMP4 in heart failure in Figure 12.

## Discussion

This study is based on our previous discovery that cardiac inhibitor of matrix metalloprotease (~TIMP4) when administered intraperitonealy, attenuates oxidative stress and brings down the levels of MMP9 in volume overload heart failure 2. In addition, the mRNA levels of TIMP4 are significantly reduced in volume overload heart failure 10. Hence, this study aims to find out the mechanisms of TIMP4 down‐regulation. As a result of volume overload, the heart is not able to pump out all the blood so that there is a decrease in the ejection fraction which was evaluated by echocardiography. The internal diameter of ventricles was also raised which was detected by echocardiography and represents hypertrophy. Literature states that TIMP4 goes down in heart failure and mice which are deficient in TIMP4 have a poor regeneration capacity post MI 23. Targeted overexpression of TIMP4 in cardiac tissues is cardioprotective and modifies extracellular matrix remodeling after MI 1. Another study from our laboratory demonstrates the importance of TIMP4 in cardiac contractility and instigating the differentiation of cardiac progenitor cells to cardiomyocytes 9. Tissue inhibitor of matrix metalloprotease 4 is one of the endogenous inhibitors of MMP9 and there are ample reports that suggest activation of MMP9 in heart failure 3, 8, 11, 24. We have reported that in normal heart most of the MMPs reside in the latent form and get activated in chronic heart failure 5, 25, 26. Although MMP9 is activated by cytokines, oxidative stress, infarct or neutrophil invasion, our *in situ* zymography data suggested that the MMP9 activity comes from the heart tissues. In addition, MMP2 was also actively up‐regulated as a result of the AVF heart failure model and was evaluated by in gelatin zymography. As a result, there is a shift in the MMP‐TIMP axis towards the MMP side. Since the mRNA levels of TIMP4 go down in heart failure, we hypothesize that there is epigenetic silencing of TIMP4 in terms of methylation of the promoter, histone acetylation and microRNA122a.

Although studies report that TIMP4 goes down in heart failure 23, there are no reports in heart which show the mechanism of TIMP4 down‐regulation. In this study, we observed down‐regulation of TIMP4 in the heart failure model made by AVF which was in agreement with our previous study 2, 10. Our hypothesis, that there is epigenetic silencing of TIMP4 stems from the fact that there are 7 CpG islands in the promoter region of TIMP4. These CpG islands have been reported to get methylated though in cancer studies. Dammann *et al*. have reported that TIMP4 frequently gets methylated (64%) in lung cancer cell lines 15. Similarly, in another study by Azhikina *et al*. it was reported that the promoter region of TIMP4 gets methylated in small lung cancer tissues 19. In agreement with these studies, in this study also we observed methylation of the CpG islands which was confirmed by sodium bisulphite treatment of the genomic DNA isolated from the WT and AVF mice followed by MS‐PCR, HRM analysis and sequencing. Apart from methylation, we also evaluated other factors which contribute to epigenetic silencing such as H_3_K9Ac, HDAC 1, DNMT 1 and microRNA122a. Although we could not observe any change in the HDAC 1 protein, we evaluated the acetylation of histone 3 at lysine 9 by ChIP analysis. This suggested that there is decrease in acetylation of the histone at the TIMP4 promoter machinery which contributes to a decrease in transcription. Similar to our study, Qin and Han have reported impaired histone acetylation at the promoter region of matrix metalloproteases which leads to their silencing 20. Qiao *et al*. 27 have reported that there is down‐regulation of histone acetyltransferase in cardiac hypertrophy, which means a decrease in histone acetylation in heart failure. When the histone acetyltransferase is overexpressed in mice, it blunts cardiac hypertrophy. Similarly, Papait *et al*. 28 have reported that a decrease in H3K9ac was significantly associated with transcription repression in trans‐aortic‐constriction cardiomyocyte. Although these reports evidence a decrease in histone acetylation with heart failure, there may be other factors like HDACs which contribute to decrease in histone acetylation. In this study, we observed only H_3_K9Ac and observed that in part, it contributes to silencing of the transcription machinery. We did not observe any change in the HDAC1.

When we evaluated the expression of DNMT1, we found elevated expression in the AVF heart tissues. Although not in heart failure, but in hyperhomocysteinemia which is a cardiovascular risk factor, we have reported that the expression of DNMT1 goes up in cardiomyocytes 22. In a similar study by Chavali *et al*. from our laboratory, we reported elevated expression of DNMT1 in diabetic cardiomyocytes, diabetes being the major cardiovascular risk factor 29. The authors evaluated the expression of DNMT3a and 3b along with DNMT1, however, they observed significant change in DNMT1 as compared to DNMT3a and 3b. This suggests that DNA methylation may not be directly associated with DNMT1, but DNMT3a and 3b may also contribute to DNA methylation as well. In agreement with this study, we observed increase in DNMT1 which contributes to DNA methylation of the TIMP4 promoter region. Altogether, the DNA methylation and histone acetylation results indicate towards repression of the transcription machinery particularly, for the TIMP4 region. We observed in one of our studies that when TIMP4 is up‐regulated due to overexpression in cardiomyocytes, the expression of mir122a goes down 9. In the present study also, we evaluated the expression of mir122a in both WT and AVF mice and observed that there is a negative correlation between these two, though we did not evaluate if mir122a directly regulates TIMP4. We also evaluated mir29b and mir455‐5p which have been shown to regulate MMP9 6. We observed down‐regulated expression of these two microRNAs in AVF mice as compared to WT mice. Since mir29b and mir455‐5p regulate the expression of MMP9, the high MMP9 levels in AVF mice can be partially attributed to these two microRNAs. Our results suggest that methylation of the CpG islands in the TIMP4 promoter region along with mir122a contribute significantly to down‐regulate the expression of TIMP4. In conclusion, our data clearly indicated that there is epigenetic silencing of TIMP4 in heart failure and strengthens the concept that if administered exogenously or overexpressed in the heart tissue, it can contribute significantly to cardioprotection.

## Conflict of interest

The authors claim that there are no conflicts of interest.
